# Supplementary material for: Phenotypic plasticity promotes recombination and gene clustering in periodic environments
Source: Nat Commun. 2017 Dec 11;8:2041. doi: 10.1038/s41467-017-01952-z (PMC5725583; doi:10.1038/s41467-017-01952-z)
Supplement: Supplementary file 1 — Supplementary Information [file 41467_2017_1952_MOESM1_ESM.pdf]

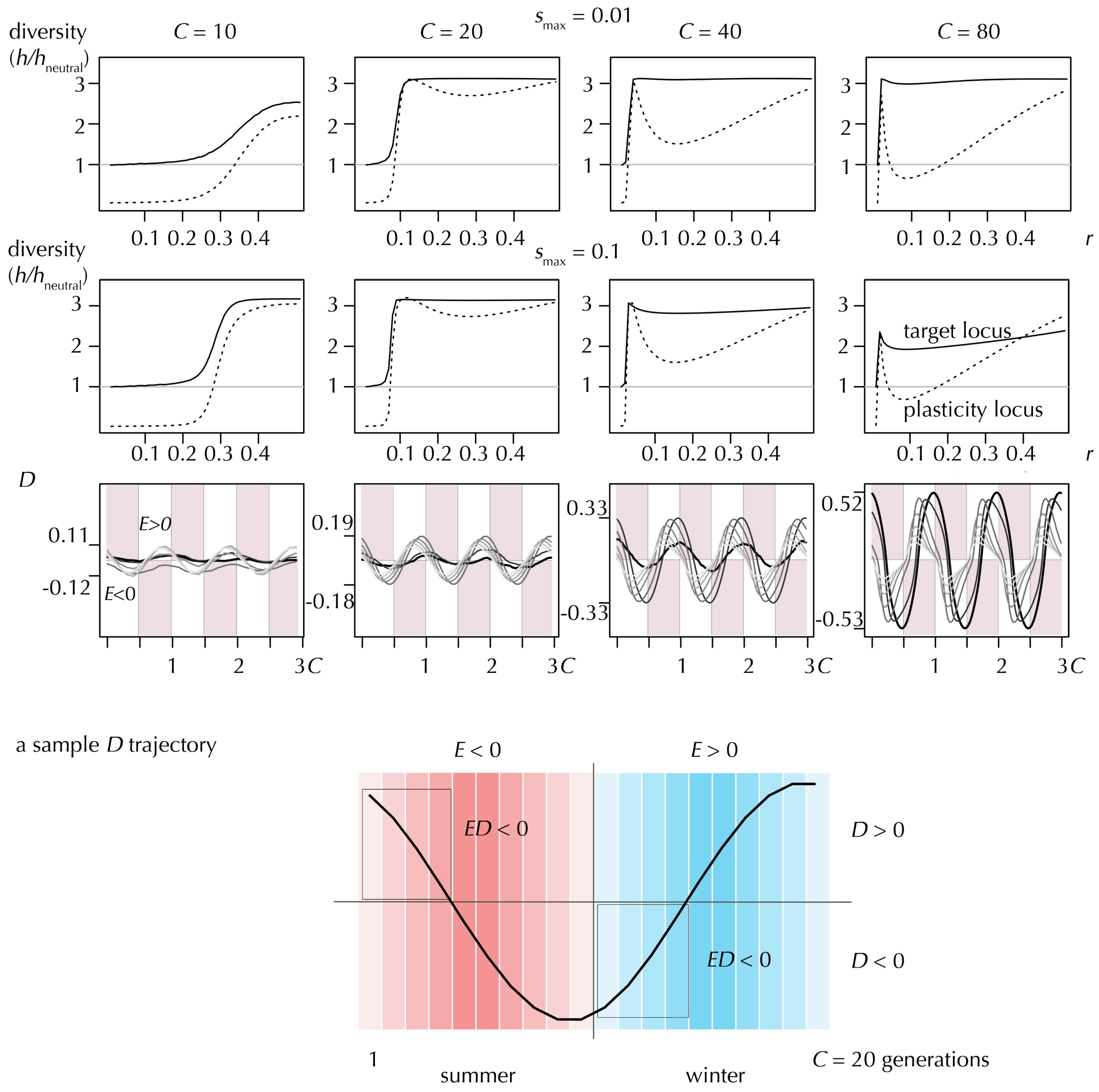

Supplementary Figure 1. Genomic storage generates both balanced polymorphism and cycling linkage disequilibrium, as necessary for the evolution of recombination. The top two rows show the levels of heterozygosity ($h = 2f_a(1\text{-}f_a)$, where f_a is the frequency of the *a* allele) relative to heterozygosity expected under neutrality at the target locus (solid line) and the plasticity modifier locus (dotted line) across the range of recombination rates [0, 0.5]. The patterns of diversity are similar in the regimes of weak selection (top row, $s_{max} = 0.01$ and $N = 10^6$) versus strong selection (second row, $s_{max} = 0.1$ and $N = 2.5\times10^4$), for all periods of environmental fluctuations (C = 10, 20, 40 and 80

generations), and with recurrent mutation rate $N\mu = 0.1$ at both of the loci. The third row shows normalized linkage disequilibrium patters ($D = D_{\text{observed}}/D_{\text{max}}$[1], with D > 0 signifying an excess of *ma*/*Md* haplotypes compared to expectation) for recombination rates of 0.01, 0.03, 0.1, 0.2, 0.3, 0.4, or 0.5 (ranging from darkest to lightest shade of grey) in the regime of strong selection. The bottom row presents an example *D* trajectory over the course of two seasons forming one cycle ($C = 20$ and recombination rate 0.1 in the regime of strong selection). At the beginning of the "summer" season, epistasis is negative ($E < 0$, meaning $w_{am} < w_{aM}$ and $w_{dM} < w_{dm}$). Negative epistasis selects for $D < 0$, in order to reduce the proportion of *am* and *dM* haplotypes in the population, and so the *D* trajectory starts to plummet. However, since the change in sign of *D* lags behind change in sign of *E*, there is a period of time (lasting four generations, in this example trajectory) with $D > 0$ and $ED < 0$. These conditions favor an increase in the recombination rate, since recombination uncouples overrepresented *am* and *dM* haplotypes. At the middle of the cycle, when the season changes to winter, *E* sharply changes sign, because $w_{am} > w_{aM}$ and $w_{dM} > w_{dm}$. Yet, *D* is now negative for the first four generations of this season, so that $ED < 0$ during this period of time, which again favors recombination. These cyclical dynamics, which exhibit sign changes in *E* followed by lagging sign changes in *D*, occur over the full range of parameters shown in the third row. The periods of time with $ED < 0$ are indicated in shaded area in the third-row; during these periods the recombination rate will be selected to increase in a population. (For all panels in this figure the recombination rate is assumed intrinsic and does not evolve.)

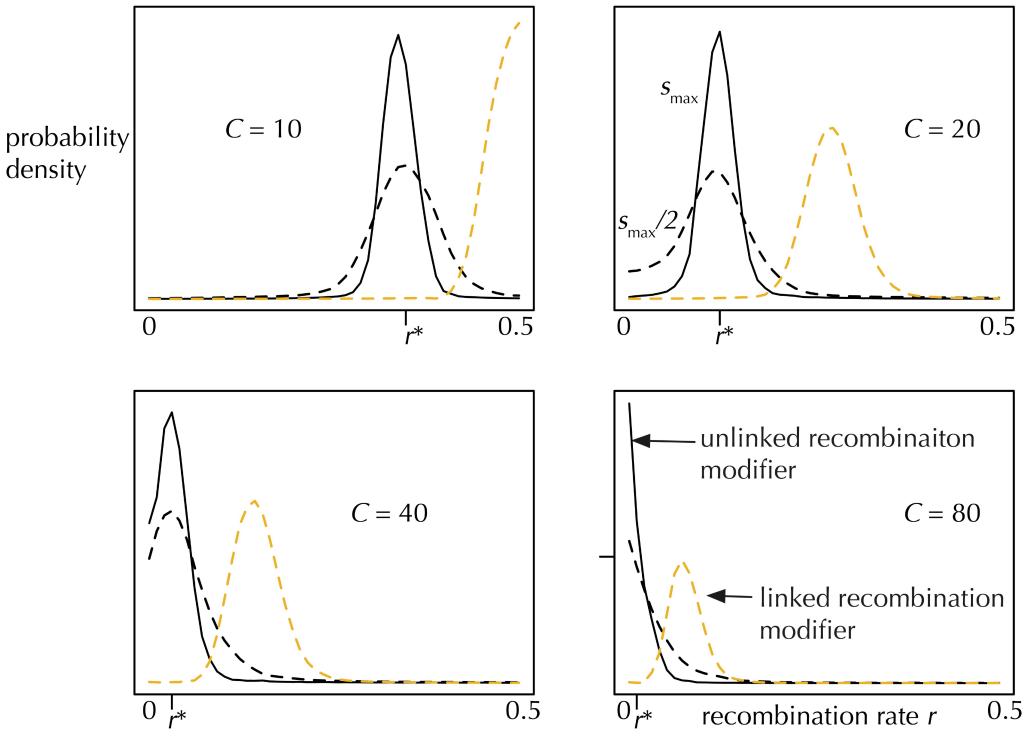

Supplementary Figure 2. Stationary distribution of the recombination rates between the plasticity and the target locus. We assume a population of $N = 25000$, with $C = 10$ and $s_{max} = 0.5$, $C = 20$ and $s_{max} = 0.25$, $C = 40$ and 80 and $s_{max} = 0.15$ (broken line indicates $s_{max}/2$) assuming the rate is coded by a recombination modifier that is unlinked (black) or linked (with $R = 0.01$, gold) to the plasticity-target sequence. $N\mu = 0.1$. The distribution is measured over the $100N$ generations past a burn-in period of $100N$ generations (after which the distribution stabilized). A tick mark on the horizontal line points to the ES recombination rate (r^*) obtained in the deterministic stability analysis with $s_{max} = 0.1$. The tick mark on vertical line in bottom right panel indicates the height of the vertical lines in other panels.

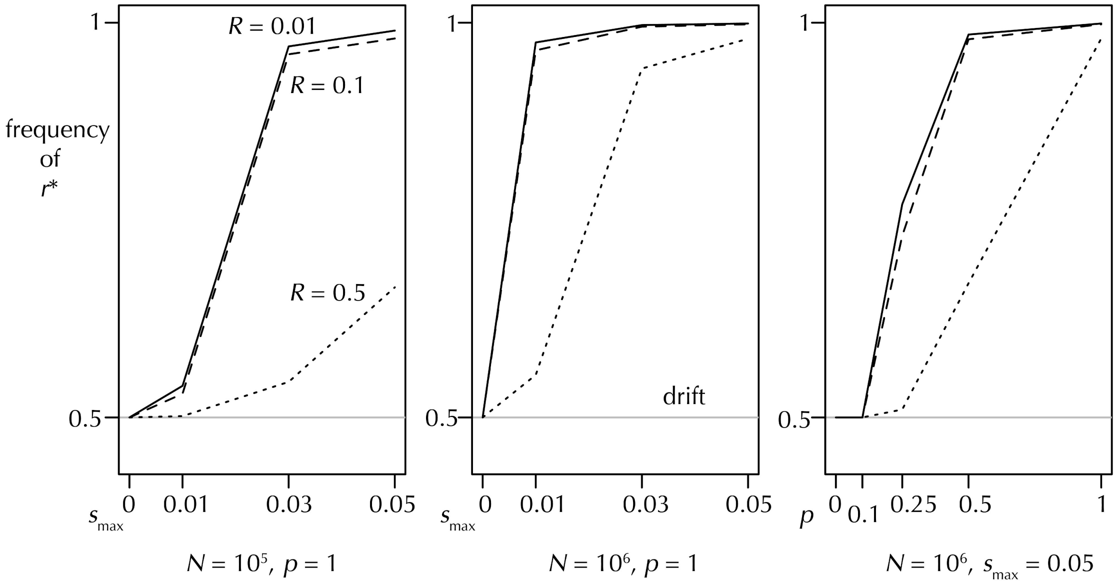

Supplementary Figure 3. **Evolution of recombination rates is more likely under larger population size, stronger plasticity effect, or when the recombination modifier locus is linked to the plasticity-target sequence**. The figure shows the equilibrium frequencies of the ES rate, which was obtained by stability analysis for each parameter combination, in initially non-recombining population under the genomic storage effect. $C = 20$, $N\mu = 0.1$ in all panels, while $p = 1$ with $N = 10^5$ and 10^6 with varying s_{max} in the first two panels; and $N = 10^6$, $s_{max} = 0.05$ with varying p in the third panel. The frequency is measured at equilibrium, after the $100N$ generations of burn-in, assuming no linkage ($R = 0.5$), weak ($R = 0.1$), or strong linkage ($R = 0.01$) between the recombination locus and the plasticity-target sequence. 40000 simulation runs were conducted for each parameter combination.

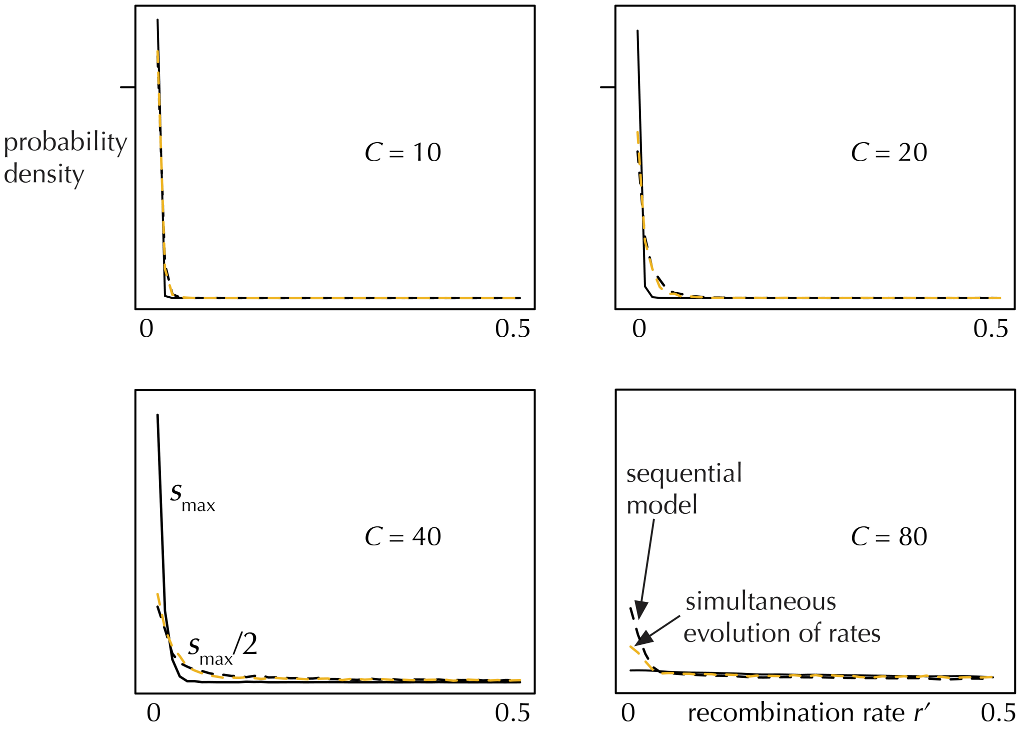

Supplementary Figure 4. Stationary distribution of the recombination rates between the two co-modified target loci. We assume that the initial target locus stars at the optimal distance to the plasticity modifier (sequential model, in black), or that both the recombination rate between the plasticity modifier and the initial target locus (due to linked recombination locus with $R = 0.01$), and between two target loci (due to unlinked recombination locus, R = 0.5), given in gold, evolve simultaneously. $N = 25000$ and $C = 10$ with $s_{max} = 0.25$, $C = 20$ with $s_{max} = 0.125$, $C = 40$ and 80 with $s_{max} = 0.075$ for each of the target loci (broken line indicates $s_{max}/2$), with $N\mu = 0.1$. The distribution is gathered over the 100N generations past the 100N generations of burn-in period (after which the distribution stabilized). The tick marks on vertical lines in top panels indicate height of vertical axis in bottom panels.

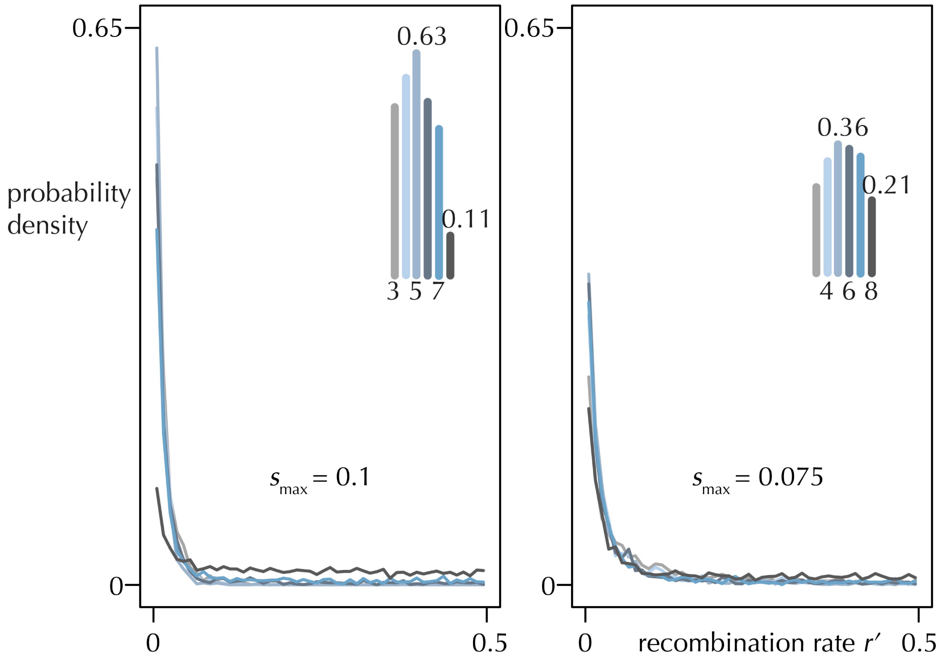

Supplementary Figure 5. Stationary distribution of recombination rates between a newly mutated target locus and the previously formed cluster of target loci. The total cluster size is ranging from three and eight loci. Here, $N = 25000$, $C = 20$, with $s_{max} = 0.1$ or 0.75 at each of the target loci, with $N\mu = 0.1$. The relative frequencies of tightly linked clusters (recombination rate < 0.01) are given in the legend. The distribution is measured over $4N$ generations following a $100N$-generations burn-in period.

Supplementary References

1. Lewontin R. C. The interaction of selection and linkage. I. General considerations; heterotic models. *Genetics* 49: 49 – 67 (1964).
